# Labor exploitation in the Italian agricultural sector: the case of vulnerable migrants in Tuscany

**DOI:** 10.3389/fsoc.2023.1234873

**Published:** 2023-10-04

**Authors:** Caterina Francesca Guidi, Fabio Berti

**Affiliations:** Laboratorio sulle Diseguaglianze, Department of Social, Political and Cognitive Sciences (DISPOC), University of Siena, Siena, Italy

**Keywords:** asylum seekers, migrants, labour exploitation, vulnerability, agriculture

## Abstract

Labor exploitation of agricultural migrant workers is a well-documented phenomenon by investigations and field research in several Italian regions, both in the North and the South. Despite the agri-food excellencies of the “Made in Italy” brand being a source of pride for Italian entrepreneurship, including the viticulture sector, evidence shows that many of these products are the result of different levels of illegal recruitment and labor exploitation. In this article, the authors analyze the impact of recent waves of vulnerable migrants entering the Italian labor market and present the results of a qualitative field research, conducted in Tuscany between 2021 and 2022. Through 60 interviews with exploited migrant workers and 40 interviews with relevant stakeholders, the authors focus on the recruitment process of vulnerable migrants into the agriculture sector and the labor conditions granted to them regardless of their particular migratory status. The article concludes with the analysis of the peculiarities of the Tuscan case study, characterized by the presence of a legal system of labor exploitation.

## 1. Introduction

The Italian agricultural sector is characterized by three distinct traits: high dependency on a foreign workforce, the precarity of the contracts linked to the seasonality of work, and a still significant share of undeclared work. As found by previous research, these traits are prevalent in European Union (EU) countries (Palumbo and Corrado, 2020; Kalantaryan et al., 2021) and, in particular, in Mediterranean agriculture (Caruso, 2016; Avallone, 2017; Papadopoulos et al., 2018; Rye and Scott, 2018; Corrado and Caruso, 2022). Sociodemographic dynamics – such as the shrinking and aging population and urbanization processes –together with low wages and the scarce attractiveness of agriculture have made this sector highly dependent on the foreign workforce. In 2020, agricultural workers in Italy amounted to 1,036 million, 34.5% of them extra-EU and 10.8% intra-EU migrants (Casella, 2021). Agricultural production – e.g., horticulture, olive growing, and viticulture – requires large numbers of workers available for limited periods of time: this specificity of working seasonally, as found in the Mediterranean basin (Hoggart and Mendoza, 1999; Labrianidis and Sykas, 2009; Kasimis and Papadopoulos, 2013; Gertel and Sippel, 2014), can only be met through the use of migrant workers. Fixed-term contracts (OTD^1^) constitute a privilege: 86% of Italian workers hold contracts of this category and 94% of migrant workers (Casella, 2021). In addition, irregular work in agriculture has been growing steadily over the last 10 years, reaching a value of 24.4%. This is almost double compared to the economy as a whole (12%) (Istat, 2020). In 2021, there were around 230,000 workers employed illegally in the Italian agricultural sector (Carchedi and Bilongo, 2022).

Tuscany is an Italian region with a long agricultural tradition. After the end of World War II, agriculture was radically transformed: producers specialized (e.g., viticulture in Chianti) and production grew. This change translated into an increased demand for an employed workforce, replacing a model based on self-exploitation and family labor. In recent times, the employed workforce grew from 24.2% in 2010 to 42.2% in 2016, and the fixed-term workforce grew to 42.3%, while undefined contracts were more than halved (Irpet, 2022). In 2020, Tuscany had more than 55 thousand active workers: almost 42% of them were migrant workers, and around 88% were employed as OTD (Casella, 2022).

**Figure 1:**
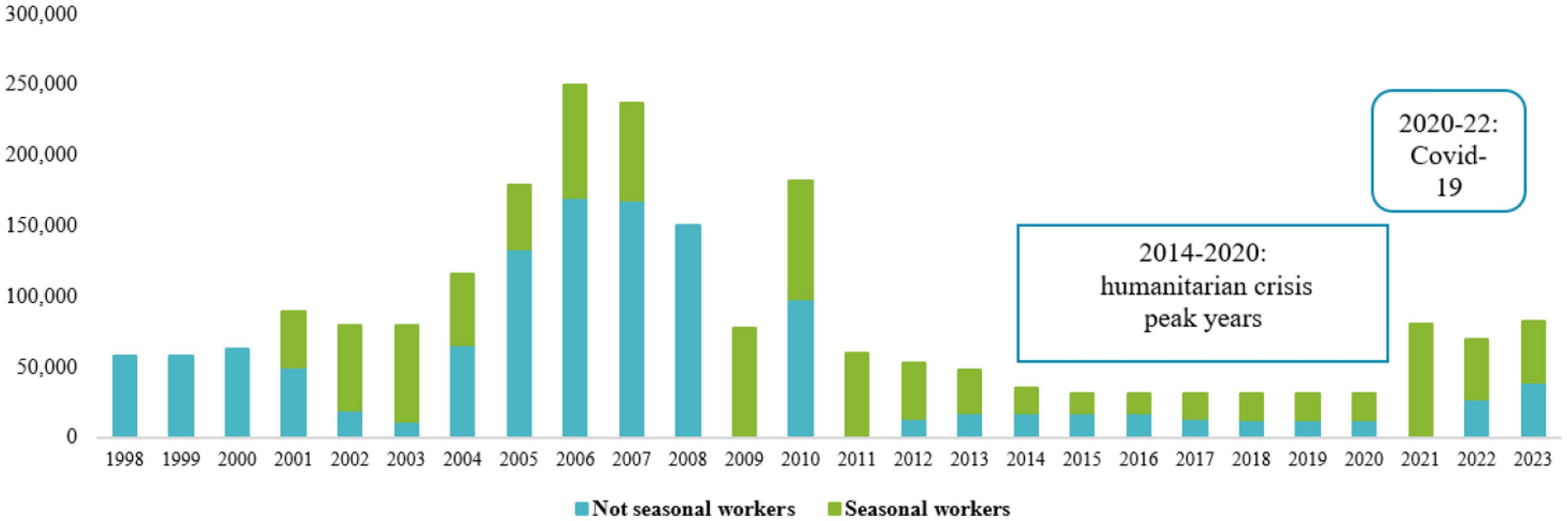
Flow decrees trend, 1998–2023 (thousands). Source: Authors’ elaboration on Italian Ministry of Interior (2023).

The study of the composition of the agricultural workforce in Italy – and therefore also in Tuscany – cannot elude the analysis of the migration policies adopted by the country to regulate labor and the social integration of migrants. The Consolidated Immigration Act (TUI) regulates the number of migrant workers who can enter the country through quotas. The quotas are regulated by a yearly government decree – the so-called flow decree (*decreto flussi*) - which defines migrant workers’ categories shares (Corrado et al., 2018). Looking at the flow decree numbers from 1998 to 2023 (Figure 1), the Italian policies on residence permits become clearer. Due to the economic crisis, since 2011, the quotas for non-seasonal employees have been drastically reduced, while the quotas for seasonal workers have been practically halved (Corrado et al., 2018). Seasonal migrant workers, allowed to enter by law, provide a small share of the agricultural workforce, considering also that intra-EU seasonal workers – e.g., Romanians and Bulgarians – can benefit from free movement within the EU and so do not fall into the quota system. The recent growth of the share of asylum seekers has therefore counterbalanced the lack of agricultural quotas in the Italian workforce. The ongoing humanitarian crisis that hit Italy in 2011 intensified in terms of the number of migrant arrivals since 2014, especially from Sub-Saharan African countries, bringing thousands of asylum seekers looking to improve their living conditions despite often being victims of dangerous border crossings. For those migrants, the only way to legalize their arrival in the country is by applying for asylum under the international protection status or thanks to special measures, such as those provided under the *North Africa Emergency* decree^2^ and subsequent emergency decrees (Corrado et al., 2018). In the last years, the emergency produced by the COVID-19 pandemic has accelerated the administrative and bureaucratic emergency governing practices for migration management in the country (Dal Zotto et al., 2021).

**Figure 2:**
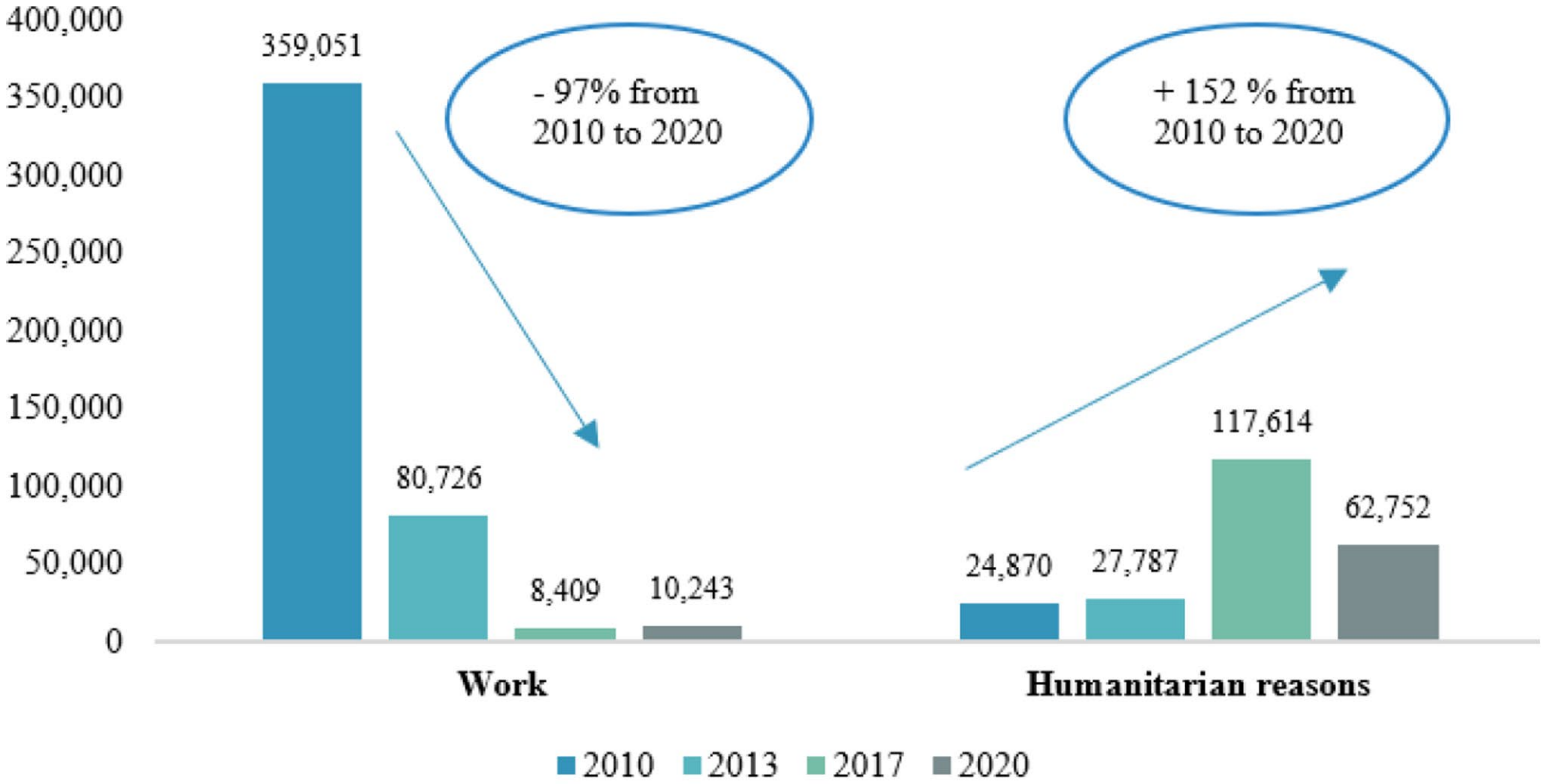
First permits for work and humanitarian reasons, 2010–2020 (thousands). Source: Authors’ elaboration on Eurostat (2023).

These arrivals have determined what Dines and Rigo (2015) first defined as the “*refugeeization*,” and what Omizzolo (2020b) and Caruso (2022) later called “*profughizzazione*”,^3^ of specific segments of migrant work, thanks also to the introduction of law no. 142/2015, according to which asylum seekers can work 60 days after they lodge their asylum application. The Italian state has, therefore, increasingly endorsed the presence of asylum seekers and holders of international protection as low-skilled workers in agriculture under conditions of precariousness and fragility. This becomes clearer when analyzing the trends of work permits together with those released for humanitarian reasons by Italy: in relative terms, while the first diminished by 97 per cent from 2010 to 2020, the other increased by 152 per cent, considering the same time span (Figure 2). The use of housing facilities - such as first (CAS) or second reception facilities (SAI)^4^ originally put in place to deal with “emergency” situations during migrant arrivals peaks – has become a constitutive component of migrant workers’ exploitation. As shown by Semprebon et al. (2017), the emergency management of seasonal migrant workers is the duty of the wider set of Italian local, regional, and national bodies, with no difference between the North and South.

Labor exploitation is the abuse, whether direct, brutal, or less obvious, of people in the workplace for profit: according to the International Labour Organization (ILO), forced labor is “*all work or service which is exacted from any person under the threat of a penalty and for which the person has not offered himself or herself voluntarily*” (ILO, 1930). People are exploited in many different ways, including having wages deducted at source, having wages and paperwork controlled by another person, being forced to work long hours without breaks, and being subjected to poor workplace health and safety through physical violence or psychological and physical abuse. In Italy, law no. 199/2016^5^ explicitly punishes all these forms of labor exploitation in agriculture by amending the criminal code (art. 603bis), targeting both abusive gang masters^6^ and employers who take advantage of workers, but often its enforcement can be difficult (Corrado and Caruso, 2022). Until recently, these forms of labor exploitation were considered a legacy of the past and the *caporalato* as exclusively prevalent in the southern parts of Italy (Perrotta, 2015; Caruso, 2016; Omizzolo, 2019). Yet field research, journalistic inquiries, and judiciary investigations have shown that Tuscany is not free from these forms of exploitation, although they can present themselves in more blurred forms, making them harder to prosecute (Oliveri, 2016; Carchedi, 2018, 2020; Cagioni, 2020; Mangano, 2020; Santoro and Stoppioni, 2020; Berti et al., 2023). Labor exploitation depends on multiple and intertwined factors. The mixture of agricultural sector labor conditions and the migration policies adopted by the country allow the labor exploitation of some of the most vulnerable groups of migrants, such as asylum seekers and holders of international protection, as will be shown by this study.

The article presents the results of an analysis of the labor exploitation of vulnerable migrants in the Tuscan agricultural sector: the analysis intends to further explore if their vulnerability is functional to the functioning of the agriculture labor market. After tracing the methodological framework, the article outlines the basic framework of the socio-economic conditions and the level of consciousness of the migrants interviewed, focusing attention on their labor conditions and exploitation. Given their legal status, vulnerable migrants should receive protection from the State, which often pushes them to accept illegal working conditions through the role played by reception facilities workers. As shown in the final part of this article on the Tuscan case study, this labor exploitation is characterized by the presence of a so-called “legal system of exploitation” (Oliveri, 2018).

## 2. Materials and methods

This article presents the research carried out within the *Demetra* project, financed by the Ministry of the Interior through the FAMI (*Fondo Asilo*, *Migrazione ed Integrazione*) fund, aiming to contrast labor exploitation in the Tuscan agricultural sector. Within this project, which had as its objectives the revealing of work exploitation cases and the reintegration through legal paths of exploited workers, the researchers of the Laboratory on Inequalities of the University of Siena were able to carry out a study aimed at analyzing the characteristics of the exploitation of migrant labor in agriculture in Tuscany (Berti et al., 2023) and the specificities of the Tuscan case of study compared to other Italian regions.

The social research was carried out using different qualitative methods, in particular semi-structured interviews aimed at different types of interviewees: 85 asylum seekers or holders of international protection, employed in the Tuscan agricultural sector, were interviewed in-depth. In this article, the focus will be on 60 out of the 85 people interviewed: those who are asylum seekers, holders of international protection, and “special cases,” i.e., those toward whom the legal discipline reserves a presumption of “vulnerability,” as explained above.

The interviews were conducted by social workers belonging to cooperatives and NGOs, as part of the Demetra project, involved in the anti-trafficking system in Tuscany. The interviewed migrants had either contacted the regional anti-trafficking system for the first time or were already known to them: starting from them, the successive interviewees were identified through the snowball method. The semi-structured interview consisted of four sections dedicated to the reconstruction of the migration experience, working conditions, housing situation, and the migrant’s health status. A preliminary interview training for the interviewers was carried out by the Laboratory on Inequalities team, who also shared the construction of the interview outline and conducted continuous monitoring to verify that a plurality of cases and characteristics of the interviewees were taken into account. In relation to the objectives of the research, the migrants interviewed were addressed through informal contacts or network relationships of the interviewers. Male and female migrant workers were addressed at their housing facilities (e.g., CAS, SAI, private apartments) since they do not always work where they live, often moving from one province to another for work-related reasons. The interviews were divided taking into account the three large healthcare areas (*Area vasta*), which are used to define the administrative boundaries of the Tuscan system: the interviews were collected for *Central Tuscany* – provinces of Florence (8 interviews), Prato (2), and Pistoia (8), *South-East Tuscany* – provinces of Arezzo (13), Siena (8), and Grosseto (14) - and *North-West Tuscany*, provinces of Lucca (13), Massa-Carrara (6), Pisa (5), and Livorno (8).

Most of the migrants showed low fluency in Italian: in some cases, the presence of cultural mediators was needed for their interviews in order to build a more meaningful and reliable interaction with the interviewees.

Sixty interviews with vulnerable migrants and asylum seekers who work or have worked in agriculture were collected. These migrants were employed especially in three sectors - olive growing and viticulture (32 people), fruit and vegetables (16), and horticulture (8). Some people (4) were employed in other residual sectors, such as fishing, livestock, or forestry, but many declared to have had different experiences in various agricultural sectors. Most of the respondents (54) were men, while only six were women. At around 29.2 years, the average age was low. This is probably connected to the strenuous nature of the work and the fact that work in agriculture is often the first step in accessing the job market. Just over half of the interviewees arrived in Italy less than 5 years ago: 94% came from Sub-Saharan Africa (in particular, from Nigeria, Gambia, Senegal, Mali, and Côte d’Ivoire) and 6% from the Asian continent (from Pakistan and Bangladesh).

In addition to interviews with exploited workers, the research group carried out 40 interviews with relevant stakeholders, divided into 5 different categories: institutions and governmental bodies, entrepreneurs and large-scale retail trade, trade union representatives, trade association representatives, and other actors, including researchers, activists, and international organization representatives. In this case, a semi-structured interview track was used, aimed at investigating the knowledge or perception of foreign workers in agriculture and their living and working conditions, of the labor exploitation and illegal recruitment system in the region, and of the effectiveness of the contrasting measures and policies adopted in this field.

All the primary materials collected were analyzed thanks to *ATLAS.ti*, a specialized software for text analysis, through the regrouping of quotations under specific codes decided ex ante. All this field activity took place between June 2021 and January 2022 during the COVID-19 pandemic. The health crisis impacted partially on the fieldwork: all the interviews with vulnerable migrants were conducted in person, thanks to personal protective equipment, while some of the interviews with the 40 relevant stakeholders were conducted online.

## 3. Results

### 3.1. The vulnerability characteristics of interviewees

In the European legislation, the definitions “vulnerability” or “special needs” remain fragmented in various directives, and so their transposition into the Italian legislation has not been an exception (Di Martino, 2018). A list of “vulnerable” categories is drawn up in the Italian legislative decree no. 142/2015, which is the transposition of European Directives no. 33 and 32 of 2013 on reception conditions and the asylum procedure. That list has been evolving to reflect the expansion of vulnerability categories along with the evolving migratory context. The term “vulnerable” can be used both for special asylum seekers and for those in conditions of need (Carnassale et al., 2021), as is done in our analysis.

Analyzing the interviews in depth, most of the migrant workers interviewed had a legal status, but this does not appear to be a guarantee for avoiding illegal working conditions, as confirmed by some relevant stakeholders.

We have verified that, compared to few years ago, the percentage of people without a residence permit is certainly a minority compared to those who have the title. Even if it is fragile - from the asylum request to article 22 [eds. of TUI]- instead of a work permit, however, the people completely without a residence permit are few compared to the number of people involved in the phenomenon. (man and woman, institutions and governmental bodies)

Looking at their documents, more than half (34 people) were asylum seekers - first instance or waiting for appeal, twenty-one obtained one of three forms of international protection - asylum, humanitarian, or subsidiary protection, while 5 people declared to have a permit for “special cases.” Besides the legal status, the nationalities of all the vulnerable migrant workers confirmed the “*refugeeization”* effect of the agricultural workforce: they do not correspond to the main Tuscan foreigner communities, represented by Romanians (17.4%), Chinese (17.3%), Albanians (13.9%), Moroccans (6.6%), and Filipinos (3%) (Istat, 2022).

Half of the interviewees were guests in the government reception system: more than half (33) lived in CAS, and only 4 people lived in SAI facilities. On the other hand, 11 lived on their own, often renting an accommodation shared with other people. It was not possible to thoroughly investigate the conditions and quality of housing: for them, the home was often shared with more than four/five people, but no further information on the level of overcrowding was released. Some interviewees (4) reported that they found a home through the employer, paying the rent to them or to the owner, who was often a friend or relative of the entrepreneur. Some workers (8) lived in the facilities of a church in the province of Pistoia.

In the majority of cases, the migratory experiences started from conflicting or economically difficult family situations, such as inducing migrants to undertake a very difficult, long, and risky journey: often, they arrived in Italy after various experiences of exploitation and abuse, especially when passing through Libya.

When I was in the Ivory Coast, I worked as an apprentice carpenter. I didn't get along with my boss; if I didn't understand things, he treated me badly [..]. I had friends who traveled towards Europe and motivated me to leave the country. One day, I decided to try my fate too and left to look for a better job. After eight months in Algeria, I went to Libya. It was worse there. I was there for three months. We were many boys; Libyans came looking for people to make them work. Then, in the evening, when it was time to pay, they threatened us with guns and didn't pay us. You couldn't do anything; it was like forced labor. Many kids have lost their lives like this. I was afraid of taking risks. Sometimes they gave me food … so after three months, I arrived in Italy in 2017. (man, Côte d’Ivoire, viticulture sector)

As to those with “special cases” permits, not all their interviews disclosed the elements of vulnerability for which their permits were issued, although in line with the rest of the interviews, most declared that they had to flee Libya.

I came to Libya because it was said that there is money, work there. When we were in Africa, a lot of people said that when you get there, you can work, do that, do that one, understand? If it had been as they said [ed. laughs], I would not have come here! For real! I wanted to stop there, but when I found the war there, my friends told me to go, because everyone didn't want to go back [ed. home] … do you understand? That's it, that's why I'm here, do you understand? (man, Senegal, fishing sector)

However, it is worth noticing that four out of five of those who had a residence permit for “special cases” continued to live under the yoke of labor exploitation, given their poor command of Italian and their urgent need for an income and to send remittances to their countries of origin.

A: What is the reason for your stay? What is written in the permit?

B: The reason is…wait...special cases. Yes, special cases. What special cases means I don't know.

A: Inside special cases there can be several things, including the fact that you have suffered severe labor exploitation and the fact that someone has harmed you is recognized, has taken advantage of you.

B: Ok! These things happen to us [ed. foreigners] all the time. (man, Senegal, fishing sector)

As underlined by Carchedi et al. (2015), once regular documents have been acquired, the exploitation circuits to which migrants are subjected are not automatically abandoned. The same victim could return to look for a job in the same environment where they had become a victim, given that often it is the only possibility of partial autonomy and independence in their living context.

Forms of labor exploitation are found even in the absence of illegal intermediation: they have been fed by the demand for low-cost work by many companies and by the social and economic vulnerability of many migrants, as revealed by the ways they have found work. They can be distinguished in three ways: those who claim to have found work thanks to the presence of a facilitator (*word-of-mouth);* those who say they have found it by presenting themselves directly to the farm (*direct action*); and those who claim to have found it through informal recruitment in meeting places - e.g., a square or bus stop but also at or through the reception facility thanks to social workers’ intermediation (*recruitment*). Many interviewees reported that they were recruited directly in the CAS or on the street: the line between informal information exchange and taking advantage of a condition of need is quite thin.

A total of 19 out of 34 asylum seekers declared that they found their work thanks to friends, 6 spontaneously introduced themselves to the farm, and 9 reported having found it through CAS or SAI workers.

They arrived here and entered the facility, but there was no one [...] so they had the opportunity to talk to someone. And then the person called me saying that there was a man who was looking for workers. Since I wanted to work, I gave him the documentation, and then he told me to be found here at 4.00 am in the morning, that someone else will come to take me [...] that's from where we left. (man, Côte d’Ivoire, fruit and vegetables sector)

A total of 13 out of 20 international protection holders declared that they found work through friends, 3 spontaneously introduced themselves to the farm, and 4 found it through recruitment. Some employers showed up at reception facilities offering a job, while others found workers at bus stops or through reception facilities employees.

A: No, I also saw many foreigners. I went on this bus, I know they maybe go to agricultural work.

B: There at 5,00 am, then?

A: Yes, at 5 am, because there is a train here at 5.30 am, then Arezzo arrives around 6 am, then I went.

B: And then you saw someone taking …

A: Someone, yes … a boy named M.

B: M.?

A: M, he saw me and said: oh, what are you looking for? I told him that I was looking for a job. He told me come and see my dad, my dad needs a worker. [...] I went there, he made me a contract for 3 months, 15 days [...] then I worked there. (man, Senegal, olive-growing and viticulture sector)

Three out of five “special cases” claimed to have found work through word-of-mouth from friends, but two of them claimed to have found it through CAS facility workers.

I started this job in 2017; I found it thanks to the cooperative worker where I lived. (man, Burkina Faso, olive-growing and viticulture sector)

This is a peculiar phenomenon considering that, in most cases, the guests of CAS or SAI found themselves in situations of labor exploitation and illegal recruitment. As recently underlined by Cortese (2020) and Casati and Pasquetti (2022), studying the rural context of southern Italy where legality is often trumped by other considerations, the legal and social inclusion of asylum seekers and migrants do not always proceed together. Given the vulnerability of one’s legal status as a migrant, certain forms of legal exploitation in the labor market increase their vulnerability, forcing them into an asymmetric position and postponing their opportunity for inclusion in Italy (even when documents have been obtained).

### 3.2. The work conditions in the legal exploitation system

More than half of the vulnerable migrant workers interviewed had a contract, but this does not represent a guarantee of greater protection and rights (Oliveri, 2018). Two types of employers have been identified: 1) the *agricultural entrepreneur*, the owner of the land and the farm; 2) the *companies* – such as *cooperatives, service company*, *and contractor company owners –* which offer services and receive subcontracts directly from agricultural entrepreneurs or other companies. Farms can source out parts of their business to external cooperatives for specialized activities (e.g., pruning) or for production phases requiring extraordinary manpower (e.g., harvesting). The subjects intermediating between the agricultural entrepreneur and workers can be of two types: the “facilitator” and the *caporale*. The “facilitator” is a loyal worker who has direct contact with the agricultural entrepreneur and helps them in the search for new workers: often co-nationals, they are people who have been living in Italy for a longer time and have become a reference point for other foreigners looking for work. These persons do not derive any advantage from their position, and their presence is not a certain indicator of illegal recruitment, as confirmed by other studies (Pugliese, 2013; Carchedi, 2020). The *caporale* has often been reported as a sort of “service agency” (Omizzolo, 2020a), because they deal with transport, organize work teams, provide accommodation, and collect useful documents for recruitment.

*The paradox of this exploitation is that there is a complicity, there is an alignment between the interests of the exploiter and those of the exploited. It is a paradox but, in many cases, this happens* [...] *the paradoxical complicity to which I alluded is determined by the fact that these people, however underpaid, miserably paid, need that poverty. If they denounce and the link that binds them to the exploiter is broken, they also lose the piece they eat and find themselves at the mercy of everything; therefore, they are attached to that piece of bread.* (man, institutional and trade union representatives)

According to the workers, almost all the companies and cooperatives that hired them committed criminal acts, except in two cases. It is precisely here that grey work starts and allows entrepreneurs to easily evade controls and sanctions: third parties not only play the role of facilitating the meeting between work supply and demand but also take the legal and economic responsibility for managing the workforce. As explained above, labor exploitation in agriculture has also been fostered by changes in labor regulations to allow for greater flexibility in labor recruitment and intermediation but also contracts regulation (Fudge and Strauss, 2014). In our case study, the workers confirmed that the most common type of contract is the OTD. Working flexibility has been widely justified by many relevant stakeholders as a necessity of the agricultural sector imposed by market needs; however, this heavily affects workers’ rights and their possibility to make stable plans for the future. The migrant workers highlighted that the OTD did not allow them to take advantage of the benefits and rights included in other types of contracts, such as holidays, illnesses, larger contributions, and seniority. Bad weather conditions, illnesses, or accidents can represent a risk to workers of not receiving their salaries, and being called to work without their salaries represents a criminal act by the agricultural company.

If I don't go to work, they don't pay me. I've never had injuries, but if I'm sick and I don't go to work, they don't pay me. (man, Nigeria, fruit and vegetables sector)

One-third of the vulnerable people interviewed reported not having a regular contract: a significant number that highlights the weight of illegal work on the regional economy. The precariousness of the fixed-term employment contract finds a counterweight in the social safety net: the agricultural unemployment allowance (NASpI). The Italian acronym stands for *Nuova Assicurazione Sociale per l’Impiego* (new social insurance for work), and it is paid at a single rate to those who have worked a minimum of 102 days during the previous 2 years in order to guarantee income continuity to OTD workers during months of forced inactivity. Although the majority of workers are aware of this support, only a small fraction actually manages to obtain it. Grey work prevents them from obtaining it, due to the lack of regularly registered worked time, and this ends up having the paradoxical effect of excluding exploited workers from a fundamental measure meant to combat poverty.

The days he gives us are few, he doesn’t pay well. Whites take so much, so much money. Blacks do not have good unemployment because the days that he marks us are few. (man, Nigeria, olive-growing and viticulture sector)

Seasonality, weather conditions, and more or less productive years are factors that determine the flexibility of the workforce: they also condition weekly rest periods and working hours (Table 1). Except in three cases, the lunch break was always granted but never paid, and days off were not guaranteed. Like the weekly rest, the daily working hours may also vary. According to the interviewees, 14 migrants reported to work between 10 and a maximum of 13 h per day; for 21 workers, the work shift could last from 8 to 10 h a day, while the normal shift was less than 8 h a day only for 16 people. Long shifts can represent both a contract violation – not paid and declared correctly in the pay slip - and a health risk for the agricultural workers who often carry out a strenuous job in prohibitive weather conditions. Fatigue and heat can make them more likely to get injured or sick.

**Table 1:** Some of the characteristics of the labor exploitation system by migratory status.

	Daily working hours			Hourly wage			Weekly rest			Means of transports			
Migratory status	Less than 8 h	Between 8 and 10 h	Between 10 and 13 h	Less than 3€	Between 4 and 6 €	Between 7 and 10 €	Yes	No	Depends	Move independently	Employer’s vehicle or reimbursement	Transport managed by intermediary	No answer
Asylum seekers	12	12	6	2	3		2	2			3	1	1
International protection holders	4	6	8	2	12	4	4	7	3	5	1	11	4
Special cases		3		1	23	5	15	11	1	13	1	14	6

Source: Authors’ elaboration on Demetra data.

The vast majority of migrant workers (38 people) declared that they received a wage between 4 and 6 euros per hour, while in nine cases, migrants received hourly wages between 7 and 10 euros, and five workers reported to be paid less than 3 euros an hour. Like other contractual conditions, the hourly wages depend on the provincial negotiation of the agricultural OTD contract: a variability that produces strong territorial disparities, even within the same region. From what emerges from the research, the minimum imposed by the provincial contract is not always respected: even in Tuscany, there are cases of payments that are between 20 and 30 euros per day, a standard that has been associated with the Southern *caporali* system (Caruso, 2016; Palumbo and Corrado, 2020; Pugliese, 2021).

I started working in agriculture at 10 years old with my father; it was better than in Italy, the job wasn't like that [ed. not ironic]! Here my back hurts, it's too hot in the greenhouses. It is an underpaid job; 4 euros is too little, it is exploitation. You do it out of patience, to get the documents, but it's not a normal job. (man, Senegal, horticulture sector)

The increased wage for holidays and overtime is almost always omitted from the paycheck and paid in cash, constituting a criminal act. According to the information collected, 20 workers confirmed that they did not have a weekly day of rest: working on Sundays was actually seen as a chance to earn a small economic surplus to be added to a very low salary. Similarly, the failure to respect the weekly day off was often not identified by workers as a serious form of exploitation.

Not all the entrepreneurs paid all the agreed salaries: 16 out of 60 vulnerable workers reported having accumulated credits toward the employer. The lack of salary affects both irregular workers and grey workers, demonstrating that the signing of a contract is not in itself a guarantee. In some cases, the owner/debtor becomes unavailable and avoids paying the majority of the sum. Not paying salaries is much easier for the owners of service companies who close their business and return to their countries of origin, thus defrauding the state and the workers without paying them contributions and salaries, respectively.

Yes, he still owes me money, but he's not there … eh … he went to Pakistan. (man, Côte d’Ivoire, fruit and vegetables sector)

Contrarily to other types of irregularities often accepted with resignation, missing payments are one of the main reasons why workers ask for justice. However, many employers know that it is difficult for a foreigner to open a trade union dispute, to report the abuse to the competent bodies, and to have the economic and social resources to initiate such an action (Omizzolo, 2019; Cagioni, 2020).

Because the money is mine and he had to give it to me, I sweated it. He told me to come back the next day, but I told him I would call the police immediately. When I told him so, he paid me. (man, Gambia, olive-growing and viticulture sector)

Some traits are typical for the Italian precarious agricultural world and affect both national and migrant workers: types of contracts, lack of weekly rest periods, unpaid meals, working days not counted in the paychecks, and so on. Non-payment reveals itself to be a type of abuse and irregularity peculiar to migrants, and it denotes asymmetries and vulnerabilities in the employment relationship. Since the interviews were carried out during hard phases of the COVID-19 pandemic, it is worth mentioning that almost all 60 migrants workers reported to not have received personal protective equipment such as masks or alcohol-based hand gel. Moreover, not one of the migrant workers reported to have received routinary medical checks or medical help when injured during the working time: one case reported to have been denied some fresh water to drink during the day. These results seem to be aligned with what was found by Holmes (2013) working with Mexican laborers in the United States: there is a link between health inequalities and the suffering of migrant workers on the field related to the structural violence of the global agricultural trade. This link normalizes the racism and symbolic violence of stereotypes and prejudices in denying health assistance to people in need.

A significant dimension of the work organization is the means of transport. Those clearly change according to the agricultural sector: vineyards often can only be reached by car, while greenhouses are easy to get to by foot or bicycle. Public transportation is often unavailable. In all, 18 of the 60 respondents moved independently if the field was nearby, mainly by bicycle or otherwise by bus or train. Meanwhile, 5 respondents reported traveling with the agricultural entrepreneur’s vehicle or receiving a reimbursement for the costs of transport or the train tickets from their employer. On the other hand, 26 reported that their transportation was managed directly by the intermediary. Commuting time is unpaid and represents an indirect cost for workers: a great sense of fatigue emerged from the workers due to the hours required to reach the workplace, added to the many working hours in the field and the poor quality of their work.

Finding work is already difficult, then the work is hard, heavy. Don't sleep during the night since cycling from Sarteano to Chianciano is long! It's all uphill...we want to work to earn some money. I'd rather do something else, but unfortunately, we have no choice (sighs) … and yes, we have to work like that. (man, Mali, olive-growing and viticulture sector)

Even though it was not explicitly asked, more than half (31 people) of the migrant workers clearly reported to have consciousness of being exploited, although few reported to know the rights they could appeal to, as demonstrated by the NASPI example.

A: Do you know what exploitation is? [ed. explains to B. what is meant by exploitation] It is important that you know what exploitation is.

B: Yes, I know. Some people, even my boyfriend, had told me that the right price would be 50, 60 euros a day, but I had no other choice.

[ed. The social worker explains that the reason for which B. accepted those conditions is due to the fact she has a daughter in Nigeria and had to pay tuition fees and school material for her. Not having found other job opportunities, she accepted that work.] (woman, Nigeria, olive-growing and viticulture sector).

In connection to studies of legal consciousness, such as those of Ewick and Silbey (1998) or Kubal (2013), it remains to be understood how much and in what way these shades of irregularities are perceived by migrants. Only 19 of the interviewed migrant workers reported having contacts with their own ethnic groups in their cities of residence, but 29 of them had contacts with local associations or NGOs from which they often received legal help. These elements, related to the domains of “rights” and “social bonds” (Ager and Strang, 2008), help to better frame the integration level of vulnerable migrants in the region.

## 4. Conclusion

This article presents the results related to the labor exploitation of vulnerable migrants in the Tuscan agricultural sector: the analysis intends to explore if their vulnerability is functional to the functioning of the agriculture labor market. Migrant workers are exposed to labor exploitation given their economic, social, and personal vulnerability, which are made up of individual, familial, structural, and symbolic elements (IOM, 2019). Scholars have demonstrated how immigration policies generate or even institutionalize the conditions under which temporary migrant workers are more vulnerable to exploitation (Lenard and Straehle, 2010; Marsden, 2012; Reilly, 2013; Zou, 2015), as in the Australian (Li and Whitworth, 2016) and Canadian (Silverman and Hari, 2016) case studies. In particular, as underlined by Rhus (2013), the institutional constraints of labor migration policy seem to influence temporary migrant workers’ agency and choices, allowing their vulnerable position to be taken advantage of.

From the field research carried out in Tuscany, some strong results emerge: the agricultural labor organization seems to function through the legal exploitation system of migrant workers in the regional agriculture sector. First of all, the abundance of workforce – especially after the so-called “humanitarian crisis” – has been mainly recruited from governmental reception centers, thus confirming the “*refugeeization*” of the workforce (Dines and Rigo, 2015; Omizzolo, 2020b). The vulnerable migrants interviewed were subjected to a double vulnerability paradox: given their legal status, asylum seekers, holders of international protection, and those with “special cases” permits should receive protection by the state, which often pushes them to accept illegal working conditions through the role played by reception facilities workers. In some cases, these choices may have been dictated by the guests’ urgent need to send back remittances - given the low daily allowance (*pocket money*) in their possession - or with the hope to help them to start their own path toward autonomy and independence. Other times, it might have been a matter of inattention toward the protection of vulnerable people during their period of stay in Italy. Following a perverse mechanism, the grey and black areas of work are encouraged by the possible decline of the first reception measures (e.g., housing, food, clothing, and a daily allowance) should the guests become independent. Although the issue of the reception measure decline is still under debate (Fiorini, 2020), the availability of a working income, equal to the national social allowance, means that the reception guests are considered autonomous and have to leave the reception facilities. Previous research has shown that Italian agricultural workers in Southern regions and local employees in reception centers do not always consider harsh working conditions and very low salaries as labor exploitation but, rather, the normal order of things (Casati and Pasquetti, 2022).

As also shown by our case study, the lack of language proficiency, the scarcity of information, the legal precariousness, the migratory debt, and the need to send remittances back home, along with widespread fear, are some of the factors that contribute to the vulnerability of migrants in the territory. As recently underlined by Obi et al. (2022) in case of Nigerian asylum seekers, the waiting time for their asylum decisions in Italy plays a further role in their wellbeing and vulnerability in accepting certain conditions for integration, such as job exploitation. Migrants in an existential situation of great fragility are then forced to choose between competitive goods such as personal safety or financial support for themselves and their families (Palumbo and Sciurba, 2015). It is, therefore, essential to adopt a global perspective on forced labor and trafficking based on human rights standards, including workers’ rights, in order to address the causes of structural vulnerability and, consequently, to challenge the reality of labor exploitation becoming a constitutive element of the work organization (Palumbo, 2022).

Secondly, a situation of formal regularity of the exploitation system has been found: labor exploitation seems to manifest itself different ways, sometimes more visible, other times more camouflaged. Severe labor exploitation episodes, threats, and abuses have been detected, even though these do not represent the norm. The Tuscan agriculture system seems rather characterized by a large formal grey area in which the exploitation dynamics materialize. These dynamics seem to be put in place by a diverse group of formally legal subjects that produce illegality within the legal framework of the agricultural work organization (Oliveri, 2018). As reported by one entrepreneur among the relevant stakeholders, “*this is a much softer hiring, this Tuscan one, compared to that of Southern Italy*”: he demonstrates more softness in terms of brutality but certainly not less violence in terms of human dignity and basic workers’ rights violations. In any case, many interviewed migrants reported having received checks, pressure, and threats during the performance of their work aimed at increasing their speed and hence profits. Working relationships seem to be formally free, but the reciprocal consensus is very apparent. This legal system of exploitation includes both the legal dimension of business, facilitated by the subcontracts mechanism, and the work organization’s dimension. This is often compounded by the use of improper contracts and, above everything else, by a real “outsourcing” of the recruitment of the exploited workforce, carried out mainly through informal channels such as word-of-mouth and ethnic networks.

The main limitation of the field work presented in this analysis is the focus on the group of migrants that is most exposed to labor exploitation. This does not give a whole perspective of the Tuscan migrant labor workforce and of the conditions suffered by settled migrants from long-term migrant communities. However, future research should focus on how migrant workers perceive their wellbeing in rural areas and in relation to other groups, as investigated in other European countries such as Germany (Glorious et al., 2020) Greece (Papadopoulos and Fratsea, 2021), Norway, and Denmark (Herslund and Paulgaard, 2021). It should be also interesting to monitor the Tuscan context to see whether, as happens in other regional contexts in Italy (Sagnet, 2017; Omizzolo, 2019), the migrant workers in this territory also organize themselves and claims their rights to obtain equal and fair work conditions.

## Data availability statement

The raw data supporting the conclusions of this article will be made available by the authors, without undue reservation.

## Ethics statement

Ethical approval was not required for the studies involving humans because the data were properly anonymized and informed consents were enough. The studies were conducted in accordance with the local legislation and institutional requirements. The participants provided their written informed consent to participate in this study.

## Author contributions

CG and FB contributed to conception and design of the study. All authors contributed to manuscript revision and read and approved the submitted version.

## Footnotes

^1^The Italian acronym OTD stands for *Operaio a Tempo Determinato*, a worker with a short-term contract.

^2^The Italian legislative decree, released on 7 April 2011, by the Prime Minister Silvio Berlusconi.

^3^The noun “*profughizzazione*” derives from the Italian term “*profugo*,” including in its definition both asylum seekers and refugees.

^4^The Italian acronym CAS stands for “*Centro di Accoglienza Straordinaria,”* while SAI stands for “*Sistema di Accoglienza ed Integrazione”* (formerly referred to as SPRAR).

^5^The Italian law no. 199/2016 “*Disposizioni in materia di contrasto ai fenomeni del lavoro nero e dello sfruttamento del lavoro in agricoltura.”*

^6^The so-called gang master system, “*caporalato*,” is an expression used to refer to illegal labor intermediation, workers’ irregular recruitment and organization, and labor exploitation (mainly) in the agricultural sector that disproportionally affect migrant workers and often lead to forced and bonded labor situations (Di Martino, 2015).
